# PHL4/411: Getting Practice on the Internet

**DOI:** 10.2196/jmir.1.suppl1.e85

**Published:** 1999-09-19

**Authors:** R G. Neville, I W. Ricketts, C McCowan, G Hoskins, I Fenton, D Newell

**Affiliations:** ^1^University of Dundee, DundeeUK

**Keywords:** Internet, General Practices, Health Care

## Abstract

**Introduction:**

Increasingly, patients are using the Internet as a source of health information, and in the near future it will become normal for patients to seek information on health care services via the Internet. Practices who wish to have an Internet presence face the awkward prospect of either designing and maintaining their own site at considerable expense in manpower and equipment or seeking an expensive commercial solution that may not be seen as a valid use of health professionals' budgets.

**Methods:**

The project was a collaborative effort between Tayside Health Board, Tayside Centre for General Practice and the Department of Applied Computing (University of Dundee), a multidisciplinary team whose members are known for their innovative research. The aim of this project was to develop, implement and maintain Internet and NHSiS Intranet Web sites for all Tayside GPs, Dentists, Community Pharmacists, Opticians and Professions Allied to Medicine (PAMS), giving our region a "national first" by displaying an entire Region's primary care web sites on the Internet and NHSiS Intranet. The Holding of information that is to be displayed on the Practice Web Sites on Tayside Health Board's Centralised database allows control of information, utilising Internet technology to allow practices around Tayside to access and control their own practice data residing on the central servers situated at Tayside Health Board.

**Results:**

User friendly software was developed to allow all of Tayside's primary care Professionals to create and maintain their own customised patient orientated Web sites. No level of Internet Programming is required to successfully use the system, the software produced will be provided with full user documentation and a support help line, with a facilitator to help with problems on site when deemed necessary. The software captured the data entered by the practices, constructed the Web site for the practice from a template design that was customised to the practice's details. The resultant Internet Documents are then published onto the servers located at Tayside Health Board (from where they can be viewed from any location on the NHSiS Intranet), whilst also being ported onto the public servers at SHOW (Scottish Health on the Web), from which they can be viewed by anyone with an Internet connection.

**Discussion:**

This project laid the foundations of good IM&T communication between Tayside Health Boardand Practices. Utilising cutting edge Internet technology, this project presented the opportunity to pilot and test novel systems of "data capture" - a natural progression from which would be the development of a paper free service administration where paper forms could be replaced by an electronic equivalent.

